# 5-Ethyl-3-(4-Fluoro­phenyl­sulfon­yl)-2-methyl-1-benzofuran

**DOI:** 10.1107/S1600536810036391

**Published:** 2010-09-18

**Authors:** Hong Dae Choi, Pil Ja Seo, Byeng Wha Son, Uk Lee

**Affiliations:** a Department of Chemistry, Dongeui University, San 24 Kaya-dong Busanjin-gu, Busan 614-714, Republic of Korea; b Department of Chemistry, Pukyong National University, 599-1 Daeyeon 3-dong, Nam-gu, Busan 608-737, Republic of Korea

## Abstract

In the title mol­ecule, C_17_H_15_FO_3_S, the 4-fluoro­phenyl ring makes a dihedral angle of 74.06 (4)° with the mean plane of the benzofuran fragment. In the crystal structure, mol­ecules are linked by weak inter­molecular C—H⋯O and C—H⋯π inter­actions. The crystal structure also exhibits aromatic π–π inter­actions between the benzene rings of adjacent mol­ecules [centroid–centroid distance = 3.629 (2) Å].

## Related literature

For the crystal structures of similar 3-(4-fluoro­phenyl­sulfonyl)-2-methyl-1-benzofuran derivatives, see: Choi *et al.* (2010*a* ▶,*b* ▶). For the biological activity of benzofuran compounds, see: Aslam *et al.* (2006 ▶); Galal *et al.* (2009 ▶); Khan *et al.* (2005 ▶). For natural products with benzofuran rings, see: Akgul & Anil (2003 ▶); Soekamto *et al.* (2003 ▶).
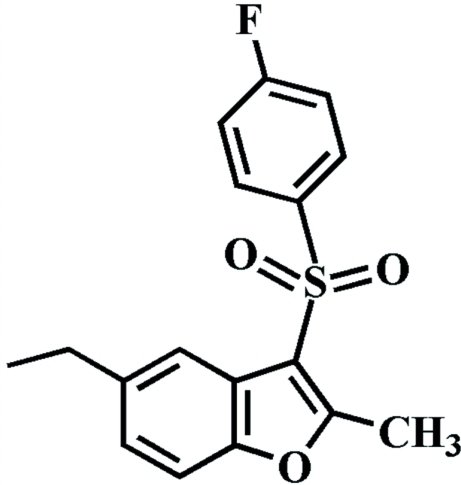


## Experimental

### 

#### Crystal data

C_17_H_15_FO_3_S
*M* *_r_* = 318.35
Triclinic, 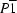

*a* = 8.0042 (1) Å
*b* = 9.7114 (2) Å
*c* = 11.3741 (2) Å
α = 66.487 (1)°
β = 82.998 (1)°
γ = 67.964 (1)°
*V* = 751.10 (2) Å^3^
*Z* = 2
Mo *K*α radiation
μ = 0.24 mm^−1^
*T* = 173 K
0.31 × 0.25 × 0.24 mm

#### Data collection

Bruker SMART APEXII CCD diffractometer
Absorption correction: multi-scan (*SADABS*; Bruker, 2009 ▶) *T*_min_ = 0.929, *T*_max_ = 0.946
13998 measured reflections
3722 independent reflections
3264 reflections with *I* > 2σ(*I*)
*R*_int_ = 0.027

#### Refinement

*R*[*F* ^2^ > 2σ(*F* ^2^)] = 0.037
*wR*(*F* ^2^) = 0.102
*S* = 1.04
3722 reflections
201 parameters
H-atom parameters constrained
Δρ_max_ = 0.33 e Å^−3^
Δρ_min_ = −0.38 e Å^−3^

### 

Data collection: *APEX2* (Bruker, 2009 ▶); cell refinement: *SAINT* (Bruker, 2009 ▶); data reduction: *SAINT*; program(s) used to solve structure: *SHELXS97* (Sheldrick, 2008 ▶); program(s) used to refine structure: *SHELXL97* (Sheldrick, 2008 ▶); molecular graphics: *ORTEP-3* (Farrugia, 1997 ▶) and *DIAMOND* (Brandenburg, 1998 ▶); software used to prepare material for publication: *SHELXL97*.

## Supplementary Material

Crystal structure: contains datablocks global, I. DOI: 10.1107/S1600536810036391/su2211sup1.cif

Structure factors: contains datablocks I. DOI: 10.1107/S1600536810036391/su2211Isup2.hkl

Additional supplementary materials:  crystallographic information; 3D view; checkCIF report

## Figures and Tables

**Table 1:** Hydrogen-bond geometry (Å, °) *Cg* is the centroid of the C2–C7 benzene ring.

*D*—H⋯*A*	*D*—H	H⋯*A*	*D*⋯*A*	*D*—H⋯*A*
C13—H13⋯O2^i^	0.95	2.40	3.3143 (17)	161
C11—H11*C*⋯*Cg*^ii^	0.98	2.66	3.491 (2)	143

Symmetry codes: (i) 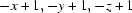; (ii) 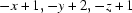.

**Fig. 1.**
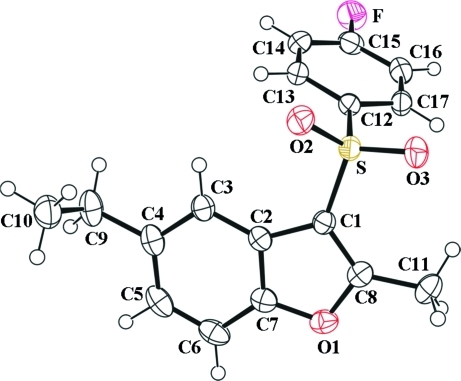
The molecular structure of the title molecule with the atom numbering scheme. Displacement ellipsoids are drawn at the 50% probability level. H atoms are presented as a small spheres of arbitrary radius.

**Fig. 2.**
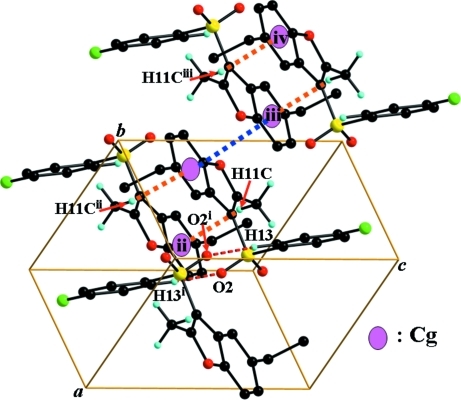
A view of the C–H···O, C–H..π and π···π interactions (dotted lines) in the crystal structure of the title compound. [Cg denotes the centroid of benzene ring C2-C7; Symmetry codes: (i) - x + 1, - y + 1, - z + 1; (ii) - x + 1, - y + 2, - z + 1; (iii) - x, - y + 2, - z + 1; (iv) x - 1, y, z.]

## supplementary crystallographic information

### Comment

Many compounds containing a benzofuran ring show important biological properties such as, antifungal (Aslam *et al.*, 2006), antitumor and antiviral (Galal *et al.*, 2009), antimicrobial (Khan *et al.*, 2005) activities. These compounds occur widely in nature (Akgul & Anil, 2003; Soekamto *et al.*, 2003). As part of our ongoing studies of the effect of side chain substituents on the solid state structures of 3-(4-fluorophenylsulfonyl)-2-methyl-1-benzofuran analogues (Choi *et al.*, 2010**a*,b*), we report herein on the crystal structure of the title compound.

In the title molecule (Fig. 1) the benzofuran unit is essentially planar, with a mean deviation of 0.009 (1) Å from the least-squares plane defined by the nine constituent atoms. The 4-fluorophenyl ring makes a dihedral angle of 74.06 (4)° with the mean plane of the benzofuran fragment.

The crystal packing (Fig. 2) is stabilized by a weak intermolecular C–H···O hydrogen bond between the 4-fluorophenyl H atom and the oxygen of the O═ S═O unit[ C13–H13···O2^i^; see Table 1], and by an intermolecular C–H···π interaction between a methyl H-atom and the benzene ring of a neighbouring molecule [C11–H11C···Cg^ii^ ; see Table 1]. The molecular packing (Fig. 2) is further stabilized by an aromatic π···π interaction between the benzene rings of neighbouring molecules, with a Cg···Cg^iii^ distance of 3.629 (2) Å (Cg is the centroid of the C2-C7 benzene ring; see Table 1).

### Experimental

77% 3-chloroperoxybenzoic acid (381 mg, 1.7 mmol) was added in small portions to a stirred solution of 5-ethyl-3-(4-fluorophenylsulfanyl)-2-methyl-1-benzofuran (229 mg, 0.8 mmol) in dichloromethane (40 mL) at 273 K. After being stirred at room temperature for 6h, the mixture was washed with saturated sodium bicarbonate solution and the organic layer was separated, dried over magnesium sulfate, filtered and concentrated at reduced pressure. The residue was purified by column chromatography (silica gel, benzene) to afford the title compound as a colorless solid [yield 82%, m.p. 377-378 K; *R*_f_ = 0.68 (benzene)]. Single crystals, suitable for X-ray diffraction, were prepared by slow evaporation of a solution of the title compound in diisopropyl ether at room temperature.

### Refinement

All the H-atoms were positioned geometrically and refined using a riding model: C–H = 0.95 Å for aryl, 0.99 Å for methylene and 0.98 Å for methyl H atoms, with *U*_iso_(H) = k × *U*_eq_(C), where k = 1.2 for aryl and methylene H-atoms, and 1.5 for methyl H-atoms.

### Crystal data

**Table:**

C_17_H_15_FO_3_S	*Z* = 2
*M**_r_* = 318.35	*F*(000) = 332
Triclinic, *P*1	*D*_x_ = 1.408 Mg m^−^^3^
Hall symbol: -P 1	Mo *K*α radiation, λ = 0.71073 Å
*a* = 8.0042 (1) Å	Cell parameters from 6492 reflections
*b* = 9.7114 (2) Å	θ = 2.5–28.2°
*c* = 11.3741 (2) Å	µ = 0.24 mm^−^^1^
α = 66.487 (1)°	*T* = 173 K
β = 82.998 (1)°	Block, colourless
γ = 67.964 (1)°	0.31 × 0.25 × 0.24 mm
*V* = 751.10 (2) Å^3^	

### Data collection

**Table:**

Bruker SMART APEXII CCD diffractometer	3722 independent reflections
Radiation source: rotating anode	3264 reflections with *I* > 2σ(*I*)
graphite multilayer	*R*_int_ = 0.027
Detector resolution: 10.0 pixels mm^-1^	θ_max_ = 28.3°, θ_min_ = 2.0°
φ and ω scans	*h* = −10→10
Absorption correction: multi-scan (*SADABS*; Bruker, 2009)	*k* = −12→12
*T*_min_ = 0.929, *T*_max_ = 0.946	*l* = −15→15
13998 measured reflections	

### Refinement

**Table:**

Refinement on *F*^2^	Primary atom site location: structure-invariant direct methods
Least-squares matrix: full	Secondary atom site location: difference Fourier map
*R*[*F*^2^ > 2σ(*F*^2^)] = 0.037	Hydrogen site location: difference Fourier map
*wR*(*F*^2^) = 0.102	H-atom parameters constrained
*S* = 1.04	*w* = 1/[σ^2^(*F*_o_^2^) + (0.0517*P*)^2^ + 0.2601*P*] where *P* = (*F*_o_^2^ + 2*F*_c_^2^)/3
3722 reflections	(Δ/σ)_max_ = 0.001
201 parameters	Δρ_max_ = 0.33 e Å^−^^3^
0 restraints	Δρ_min_ = −0.38 e Å^−^^3^

### Special details

**Table:**

Geometry. All esds (except the esd in the dihedral angle between two l.s. planes) are estimated using the full covariance matrix. The cell esds are taken into account individually in the estimation of esds in distances, angles and torsion angles; correlations between esds in cell parameters are only used when they are defined by crystal symmetry. An approximate (isotropic) treatment of cell esds is used for estimating esds involving l.s. planes.
Refinement. Refinement of F^2^ against ALL reflections. The weighted R-factor wR and goodness of fit S are based on F^2^, conventional R-factors R are based on F, with F set to zero for negative F^2^. The threshold expression of F^2^ > 2sigma(F^2^) is used only for calculating R-factors(gt) etc. and is not relevant to the choice of reflections for refinement. R-factors based on F^2^ are statistically about twice as large as those based on F, and R- factors based on ALL data will be even larger.

### Fractional atomic coordinates and isotropic or equivalent isotropic displacement parameters (Å^2^)

**Table:**

	*x*	*y*	*z*	*U*_iso_*/*U*_eq_	
S1	0.53064 (4)	0.61417 (4)	0.69284 (3)	0.02667 (10)	
F1	0.02306 (15)	0.28696 (12)	0.93170 (10)	0.0495 (3)	
O1	0.26044 (14)	1.07835 (12)	0.57702 (10)	0.0326 (2)	
O2	0.61844 (14)	0.55702 (13)	0.59437 (10)	0.0352 (2)	
O3	0.63741 (14)	0.59481 (13)	0.79503 (10)	0.0354 (2)	
C1	0.39955 (18)	0.81537 (16)	0.61842 (12)	0.0255 (3)	
C2	0.29794 (17)	0.88735 (16)	0.49822 (12)	0.0261 (3)	
C3	0.26704 (18)	0.83239 (17)	0.40981 (13)	0.0290 (3)	
H3	0.3221	0.7221	0.4220	0.035*	
C4	0.15386 (19)	0.94248 (19)	0.30318 (13)	0.0331 (3)	
C5	0.0762 (2)	1.10491 (19)	0.28633 (15)	0.0381 (3)	
H5	0.0007	1.1788	0.2126	0.046*	
C6	0.1050 (2)	1.16199 (18)	0.37250 (15)	0.0371 (3)	
H6	0.0517	1.2724	0.3600	0.045*	
C7	0.21583 (18)	1.04904 (17)	0.47799 (13)	0.0295 (3)	
C8	0.37157 (18)	0.93495 (17)	0.66117 (13)	0.0285 (3)	
C9	0.1224 (2)	0.8853 (2)	0.20518 (15)	0.0438 (4)	
H9A	0.0020	0.9550	0.1632	0.053*	
H9B	0.1225	0.7745	0.2496	0.053*	
C10	0.2631 (2)	0.8871 (2)	0.10352 (16)	0.0450 (4)	
H10A	0.2596	0.9975	0.0562	0.067*	
H10B	0.2381	0.8462	0.0441	0.067*	
H10C	0.3828	0.8187	0.1444	0.067*	
C11	0.4355 (2)	0.9415 (2)	0.77493 (15)	0.0372 (3)	
H11A	0.4909	0.8320	0.8383	0.056*	
H11B	0.3331	1.0027	0.8127	0.056*	
H11C	0.5246	0.9942	0.7491	0.056*	
C12	0.37463 (18)	0.51746 (15)	0.76302 (12)	0.0267 (3)	
C13	0.3061 (2)	0.45940 (16)	0.69364 (13)	0.0314 (3)	
H13	0.3415	0.4729	0.6081	0.038*	
C14	0.1857 (2)	0.38180 (17)	0.75030 (15)	0.0358 (3)	
H14	0.1357	0.3425	0.7044	0.043*	
C15	0.1402 (2)	0.36313 (17)	0.87531 (15)	0.0350 (3)	
C16	0.2084 (2)	0.41710 (18)	0.94653 (14)	0.0344 (3)	
H16	0.1758	0.3997	1.0330	0.041*	
C17	0.32586 (19)	0.49745 (17)	0.88873 (13)	0.0302 (3)	
H17	0.3729	0.5387	0.9346	0.036*	

### Atomic displacement parameters (Å^2^)

**Table:**

	*U*^11^	*U*^22^	*U*^33^	*U*^12^	*U*^13^	*U*^23^
S1	0.02624 (17)	0.02837 (17)	0.02451 (17)	−0.00613 (13)	−0.00164 (12)	−0.01197 (13)
F1	0.0537 (6)	0.0503 (6)	0.0491 (6)	−0.0316 (5)	0.0016 (5)	−0.0115 (5)
O1	0.0343 (5)	0.0282 (5)	0.0366 (5)	−0.0118 (4)	0.0075 (4)	−0.0151 (4)
O2	0.0347 (5)	0.0364 (5)	0.0325 (5)	−0.0060 (4)	0.0044 (4)	−0.0186 (4)
O3	0.0327 (5)	0.0419 (6)	0.0319 (5)	−0.0118 (4)	−0.0074 (4)	−0.0135 (4)
C1	0.0258 (6)	0.0270 (6)	0.0248 (6)	−0.0096 (5)	0.0020 (5)	−0.0112 (5)
C2	0.0233 (6)	0.0285 (6)	0.0239 (6)	−0.0093 (5)	0.0032 (5)	−0.0082 (5)
C3	0.0272 (6)	0.0331 (7)	0.0258 (6)	−0.0113 (5)	0.0014 (5)	−0.0101 (5)
C4	0.0253 (6)	0.0458 (8)	0.0250 (6)	−0.0145 (6)	0.0023 (5)	−0.0093 (6)
C5	0.0264 (7)	0.0427 (8)	0.0291 (7)	−0.0079 (6)	0.0000 (5)	−0.0020 (6)
C6	0.0296 (7)	0.0283 (7)	0.0391 (8)	−0.0049 (6)	0.0051 (6)	−0.0050 (6)
C7	0.0270 (6)	0.0294 (7)	0.0300 (7)	−0.0111 (5)	0.0064 (5)	−0.0101 (5)
C8	0.0270 (6)	0.0324 (7)	0.0300 (7)	−0.0134 (5)	0.0074 (5)	−0.0151 (5)
C9	0.0389 (8)	0.0638 (11)	0.0297 (8)	−0.0213 (8)	−0.0046 (6)	−0.0146 (7)
C10	0.0487 (9)	0.0479 (9)	0.0336 (8)	−0.0118 (8)	0.0014 (7)	−0.0161 (7)
C11	0.0411 (8)	0.0454 (8)	0.0375 (8)	−0.0199 (7)	0.0083 (6)	−0.0262 (7)
C12	0.0279 (6)	0.0235 (6)	0.0256 (6)	−0.0050 (5)	−0.0036 (5)	−0.0092 (5)
C13	0.0392 (8)	0.0273 (6)	0.0268 (6)	−0.0077 (6)	−0.0049 (6)	−0.0116 (5)
C14	0.0448 (8)	0.0291 (7)	0.0369 (8)	−0.0136 (6)	−0.0068 (6)	−0.0135 (6)
C15	0.0349 (7)	0.0283 (7)	0.0375 (8)	−0.0113 (6)	−0.0043 (6)	−0.0067 (6)
C16	0.0341 (7)	0.0367 (7)	0.0274 (7)	−0.0094 (6)	−0.0014 (6)	−0.0098 (6)
C17	0.0314 (7)	0.0326 (7)	0.0264 (6)	−0.0080 (5)	−0.0042 (5)	−0.0128 (5)

### Geometric parameters (Å, °)

**Table:**

S1—O3	1.4366 (10)	C9—C10	1.512 (2)
S1—O2	1.4380 (10)	C9—H9A	0.9900
S1—C1	1.7302 (13)	C9—H9B	0.9900
S1—C12	1.7656 (14)	C10—H10A	0.9800
F1—C15	1.3542 (17)	C10—H10B	0.9800
O1—C8	1.3642 (17)	C10—H10C	0.9800
O1—C7	1.3811 (17)	C11—H11A	0.9800
C1—C8	1.3652 (18)	C11—H11B	0.9800
C1—C2	1.4496 (18)	C11—H11C	0.9800
C2—C7	1.3874 (19)	C12—C17	1.3905 (19)
C2—C3	1.3947 (19)	C12—C13	1.3909 (19)
C3—C4	1.3932 (19)	C13—C14	1.385 (2)
C3—H3	0.9500	C13—H13	0.9500
C4—C5	1.401 (2)	C14—C15	1.380 (2)
C4—C9	1.510 (2)	C14—H14	0.9500
C5—C6	1.380 (2)	C15—C16	1.375 (2)
C5—H5	0.9500	C16—C17	1.384 (2)
C6—C7	1.382 (2)	C16—H16	0.9500
C6—H6	0.9500	C17—H17	0.9500
C8—C11	1.4802 (19)		
			
O3—S1—O2	119.42 (6)	C4—C9—H9B	109.0
O3—S1—C1	109.13 (6)	C10—C9—H9B	109.0
O2—S1—C1	107.67 (6)	H9A—C9—H9B	107.8
O3—S1—C12	107.56 (6)	C9—C10—H10A	109.5
O2—S1—C12	107.33 (6)	C9—C10—H10B	109.5
C1—S1—C12	104.80 (6)	H10A—C10—H10B	109.5
C8—O1—C7	107.11 (10)	C9—C10—H10C	109.5
C8—C1—C2	107.43 (12)	H10A—C10—H10C	109.5
C8—C1—S1	126.92 (11)	H10B—C10—H10C	109.5
C2—C1—S1	125.65 (10)	C8—C11—H11A	109.5
C7—C2—C3	119.46 (13)	C8—C11—H11B	109.5
C7—C2—C1	104.60 (12)	H11A—C11—H11B	109.5
C3—C2—C1	135.93 (13)	C8—C11—H11C	109.5
C4—C3—C2	118.67 (13)	H11A—C11—H11C	109.5
C4—C3—H3	120.7	H11B—C11—H11C	109.5
C2—C3—H3	120.7	C17—C12—C13	121.14 (14)
C3—C4—C5	119.60 (14)	C17—C12—S1	118.90 (11)
C3—C4—C9	119.24 (15)	C13—C12—S1	119.93 (11)
C5—C4—C9	121.12 (14)	C14—C13—C12	119.42 (13)
C6—C5—C4	122.72 (14)	C14—C13—H13	120.3
C6—C5—H5	118.6	C12—C13—H13	120.3
C4—C5—H5	118.6	C15—C14—C13	118.15 (13)
C5—C6—C7	116.07 (14)	C15—C14—H14	120.9
C5—C6—H6	122.0	C13—C14—H14	120.9
C7—C6—H6	122.0	F1—C15—C16	117.89 (14)
O1—C7—C6	125.99 (13)	F1—C15—C14	118.60 (14)
O1—C7—C2	110.55 (12)	C16—C15—C14	123.51 (14)
C6—C7—C2	123.46 (14)	C15—C16—C17	118.15 (13)
O1—C8—C1	110.32 (12)	C15—C16—H16	120.9
O1—C8—C11	115.33 (12)	C17—C16—H16	120.9
C1—C8—C11	134.35 (14)	C16—C17—C12	119.59 (13)
C4—C9—C10	112.95 (14)	C16—C17—H17	120.2
C4—C9—H9A	109.0	C12—C17—H17	120.2
C10—C9—H9A	109.0		
			
O3—S1—C1—C8	−11.72 (15)	C7—O1—C8—C1	0.22 (15)
O2—S1—C1—C8	−142.73 (12)	C7—O1—C8—C11	179.75 (11)
C12—S1—C1—C8	103.23 (13)	C2—C1—C8—O1	−0.33 (15)
O3—S1—C1—C2	168.62 (11)	S1—C1—C8—O1	179.95 (9)
O2—S1—C1—C2	37.61 (13)	C2—C1—C8—C11	−179.74 (15)
C12—S1—C1—C2	−76.43 (12)	S1—C1—C8—C11	0.6 (2)
C8—C1—C2—C7	0.31 (14)	C3—C4—C9—C10	−86.77 (18)
S1—C1—C2—C7	−179.97 (10)	C5—C4—C9—C10	91.01 (18)
C8—C1—C2—C3	−178.43 (15)	O3—S1—C12—C17	24.49 (13)
S1—C1—C2—C3	1.3 (2)	O2—S1—C12—C17	154.16 (11)
C7—C2—C3—C4	0.01 (19)	C1—S1—C12—C17	−91.56 (12)
C1—C2—C3—C4	178.61 (14)	O3—S1—C12—C13	−153.99 (11)
C2—C3—C4—C5	0.9 (2)	O2—S1—C12—C13	−24.32 (13)
C2—C3—C4—C9	178.68 (13)	C1—S1—C12—C13	89.96 (12)
C3—C4—C5—C6	−0.9 (2)	C17—C12—C13—C14	0.7 (2)
C9—C4—C5—C6	−178.63 (14)	S1—C12—C13—C14	179.12 (11)
C4—C5—C6—C7	−0.1 (2)	C12—C13—C14—C15	−0.9 (2)
C8—O1—C7—C6	179.79 (13)	C13—C14—C15—F1	−179.79 (13)
C8—O1—C7—C2	−0.01 (15)	C13—C14—C15—C16	−0.1 (2)
C5—C6—C7—O1	−178.77 (13)	F1—C15—C16—C17	−178.93 (13)
C5—C6—C7—C2	1.0 (2)	C14—C15—C16—C17	1.4 (2)
C3—C2—C7—O1	178.81 (11)	C15—C16—C17—C12	−1.6 (2)
C1—C2—C7—O1	−0.18 (14)	C13—C12—C17—C16	0.6 (2)
C3—C2—C7—C6	−1.0 (2)	S1—C12—C17—C16	−177.84 (10)
C1—C2—C7—C6	−179.99 (13)		

### Hydrogen-bond geometry (Å, °)

**Table:**

Cg is the centroid of the C2–C7 benzene ring.

**Table:**

*D*—H···*A*	*D*—H	H···*A*	*D*···*A*	*D*—H···*A*
C13—H13···O2^i^	0.95	2.40	3.3143 (17)	161
C11—H11C···Cg^ii^	0.98	2.66	3.491 (2)	143

Symmetry codes: (i) −*x*+1, −*y*+1, −*z*+1; (ii) −*x*+1, −*y*+2, −*z*+1.
